# Distribution of HPV Genotypes Differs Depending on Behavioural Factors among Young Women

**DOI:** 10.3390/microorganisms9040750

**Published:** 2021-04-02

**Authors:** Laura Bergqvist, Ilkka Kalliala, Karoliina Aro, Eeva Auvinen, Maija Jakobsson, Mari Kiviharju, Seppo Virtanen, Joakim Dillner, Pekka Nieminen, Karolina Louvanto

**Affiliations:** 1 Department of Obstetrics and Gynecology, University of Helsinki and Helsinki University Hospital, 00029 Helsinki, Finland; ilkka.kalliala@hus.fi (I.K.); karoliina.aro@hus.fi (K.A.); mari.kiviharju@hus.fi (M.K.); seppo.virtanen@hus.fi (S.V.); pekka.nieminen@hus.fi (P.N.); 2 Department of Metabolism, Digestion and Reproduction and Department of Surgery and Cancer, Faculty of Medicine, Imperial College London, London SW7 2AZ, UK; 3 Department of Virology, University of Helsinki and Helsinki University Hospital, 00014 Helsinki, Finland; eeva.auvinen@hus.fi; 4 Department of Obstetrics and Gynecology, Hyvinkää Hospital, HUCH and University of Helsinki, 05850 Hyvinkää, Finland; maija.jakobsson@hus.fi; 5 Department of Laboratory Medicine, Karolinska Institute, SE-171 77 Stockholm, Sweden; joakim.dillner@ki.se; 6 Department of Obstetrics and Gynecology, Tampere University Hospital and Tampere University, 33100 Tampere, Finland; karolina.louvanto@tuni.fi

**Keywords:** colposcopy, human papillomavirus (HPV), risk factor, questionnaire, high-risk human papillomavirus (HPV)

## Abstract

Risk factors for the different human papillomavirus (HPV) genotypes are not well understood, although the risk of cancer is known to vary among them. Our aim was to evaluate the association of diverse behavioral and reproductive factors with genotype-specific HPV prevalence among 879 unvaccinated women aged 18–75 years referred to the colposcopy clinic at Helsinki University Hospital in Finland. Cervical swabs for HPV genotyping were collected in the first visit and assessed for 34 high-risk (hr) and low-risk (lr) HPV genotypes. Participants completed a questionnaire on behavioral, reproductive, and lifestyle factors. Differences in genotype-specific HPV prevalence were analyzed overall and in age groups using binary logistic regression. Smoking was associated with higher prevalence in HPV16 compared with other hrHPV genotypes together with decreasing age, being highest among younger women <30 years old, odds ratio (OR) 3.74 (95% CI 1.42–9.88). The later the sexual debut, the more it seemed to protect from HPV16 infection. The best protection was achieved when the sexual debut took place at >20 years of age, with an OR of 0.43 (95% CI 0.23–0.83). This association was not seen with other hrHPV genotypes. Methods of contraception seemed not to have an effect on hrHPV positivity, regardless of the HPV genotype. The genotype specific hrHPV prevalence differs, depending on behavioral factors, especially among younger women referred to colposcopy.

## 1. Introduction

Persistent infection with high-risk human papillomavirus (hrHPV) is necessary for the development of cervical precancerous lesions and cancer [1]. Among women with normal cytology, the most common HPV genotype is HPV16, but the prevalence of other genotypes varies in different geographic regions. HPV53 is the second-most common genotype in North America while it is HPV52 in Asia, whereas the second-most common genotypes in Europe and Africa are HPV31 and HPV58, respectively [2]. The prevalence of HPV18 (1.1) lists fifth globally, being the sixth-most prevalent in Europe and ninth in North America [2]. The known risk factors to acquire an HPV infection are related to sexual behavior [3], and the prevalence peaks at a young age after sexual debut [4]. The transmission of HPV between couples is extremely common [5]. A high number of sex partners is the most important risk factor for HPV positivity [5,6,7], and a recently acquired new partner increases this risk further [7]. Smoking, long-term oral contraceptive use, multiparity, and a young age at first intercourse are associated with the risk of high-grade squamous intraepithelial lesions (HSILs) [7,8] and seem to be important in the progress of cervical precancerous lesions to cervical cancer [8,9].

It is not well known, however, whether all risk factors associate with or contribute to the presence or persistence of all HPV genotypes equally. The carcinogenic potential between the different HPV genotypes varies significantly, with HPV16 being the most carcinogenic genotype [10], having a high attribution (55%) in cervical neoplastic lesions, followed by HPV18 [2]. Both HPV16 and HPV18 have recently been detected in a set of cervical intraepithelial lesions with a prevalence of approximately 61% and 39%, respectively [11].

Among women referred to colposcopy, the prevalence of HPV infection is much higher than in the overall screening population, and the HPV infection has likely persisted for a longer period, enough to cause the cytopathological changes. Our objective was to investigate the association between reproductive, behavioral, and other lifestyle-related risk factors and their association with the prevalence of the different HPV genotypes in this unvaccinated, well-screened population referred to colposcopy in Finland.

## 2. Materials and Methods

This study was part of a prospective cohort study (ISRCTN10933736) conducted at Helsinki University Hospital in Finland between January 2014 and December 2017, recruiting 1383 women referred to colposcopy according to the Finnish Current Care Guidelines [12]. All participants provided written informed consent.

Cervical samples were collected in a sample transport medium (STM, Qiagen GMBH, Hilden, Germany) for HPV DNA detection at baseline and stored at −20 °C. They were later divided into three aliquots and stored further at −80 °C. One frozen aliquot controlled for the sufficiency of each sample was sent to an HPV reference laboratory at the Karolinska Institute in Stockholm, Sweden for HPV genotyping, which was performed with the Maldi-Tof Luminex assay, as previously described in [13]. The samples were tested for 14 high-risk or possibly high-risk genotypes (hrHPV: 16, 18, 31, 33, 35, 39, 45, 51, 52, 56, 58, 59, 66, and 68) and 20 low-risk genotypes (lrHPV: 6, 11, 30, 40, 42, 43, 53, 61, 67, 69, 70, 73, 74, 81, 83, 86, 87, 89, 90, and 91).

The referral cytology results were classified according to the Bethesda system, including atypical squamous cells of undetermined significance (ASCUS), low-grade squamous intraepithelial lesions (LSILs), high-grade squamous intraepithelial lesions (HSILs), atypical squamous cells that cannot exclude HSIL (ASC-H), atypical glandular cells not otherwise specified (AGC-NOS), and atypical glandular cells favoring neoplasia (AG-FN).

All participants had a colposcopic examination with punch biopsies according to the discretion of the colposcopist. Concurrent with the colposcopic examination, the women completed a questionnaire concerning parity, recent contraception, age at first sexual intercourse, number of lifetime sex partners, practice of anal or oral sex, having had more than one sex partner (>1) in the last 12 months, termination of pregnancy, miscarriages, alcohol consumption, tobacco use, drug abuse, atopic skin, and use of vitamin D. Those who did not complete the questionnaire at the clinic were invited to participate by text messaging to fill in the same questionnaire through a web-based data collection tool Webropol (Webropol, Helsinki, Finland) designed to support data capture for research studies [14].

Because of the variation of HPV prevalence and background factors by age, we computed the prevalence percentages of all background characteristics from the questionnaire and the corresponding frequencies of all 34 HPV genotypes overall and in three age groups (<30 years, 30–44 years, and ≥45 years). The HPV genotyping results were categorized as follows: HPV16 only (HPV16), hrHPV without HPV16 (non16-hrHPV), and lrHPV. Non16-hrHPV included all high-risk HPVs except HPV16 (HPV18, 31, 33, 35, 39, 45, 51, 52, 56, 58, 59, 66, and 68). LrHPV included all low-risk HPVs, either presented as a single or multiple infections, not involving an hrHPV. Multiple HPV was defined as infection with two or more (≥2) different HPV genotypes simultaneously.

We used binary logistic regression to calculate the odds ratios (ORs) with 95% confidence intervals (95% CIs) to evaluate the association between the different risk factors and HPV group positivity (HPV16, non16-hrHPV, and lrHPV) overall, stratified for each age group. The reference group for HPV 16 and non16-hrHPV consisted of HPV-negative women and women who were positive for lrHPV (single or multiple), and for lrHPV, the reference group consisted of all HPV-negative women. We further formally tested for interaction between groups of risk factors for different HPV group positivity. Whenever statistically significant interaction was present (*p* < 0.05), we stratified the analysis according to the two relevant risk factor groups. Differences in HPV genotype prevalence among age groups were calculated using a chi-square test or Fisher’s exact test when appropriate. All statistical tests were two-sided, with *p*-values <0.05 considered statistically significant. All analyses were performed using STATA/SE 15 (StataCorp, College Station, TX, USA).

## 3. Results

Of the 1383 recruited women, the final study population consisted of 879 women who filled in the background questionnaire and for whom HPV genotyping was successfully performed. In 485 women, the questionnaire was missing, and in 19 women, the HPV test result was not available; these women were excluded. Of all 879 women, 483 (55.0%) completed the questionnaire using a web-based data collection tool and 396 (45.0%) completed the paper version. The age at sampling varied between 19–75 years with a mean age of 36.9 years (SD ± 10.5). Of all 879 women, 234 (26.6%) were under 30 years, 444 (50.5%) were 30–44 years old, and 201 (22.9%) were 45 years or older. Most of the women were referred due to abnormal cytology (94.0%); other reasons included a history of postcoital bleeding (1.0%), persistent HPV infection (1.4%), and a loop electrosurgical excision procedure (LEEP) or follow-up (3.6%).

**Figure 1:**
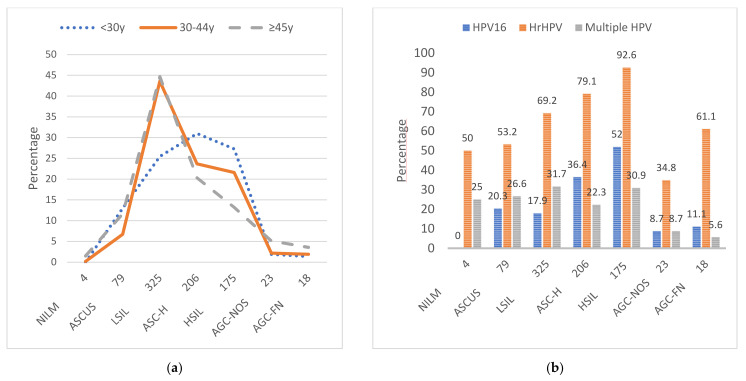
Frequencies and distributions of referral cytology by age and human papillomavirus (HPV) genotype. (**a**) Referral cytology shown by age groups (<30, 30–44, and ≥45 years). (**b**) The prevalence of HPV shown in three designated groups within specific referral cytology among all women, including only HPV16; high-risk (hr) HPVs including the following HPV genotypes: 16, 18, 31, 33, 35, 39, 45, 51, 52, 56, 58, 59, 66, and 68; and multiple HPV. Multiple HPV was defined as having two or more HPV genotypes detected simultaneously. ASCUS = atypical squamous cells of undetermined significance; LSIL = low-grade squamous intraepithelial lesion; HSIL = high-grade squamous intraepithelial lesion; ASC-H = atypical squamous cells that cannot exclude HSIL; AGC-NOS = atypical glandular cells not otherwise specified; AGC-FN = atypical glandular cells that favor neoplasia; and NILM = negative for intraepithelial lesion or malignancy.

LSIL was the most common referral cytology in the two oldest age groups, whereas HSIL and ASC-H had the highest proportion in the youngest age group (Figure 1). In women under 30 years of age, only repeated LSIL within 12 months warranted referral to colposcopy. The most prevalent HPV genotypes within high-grade cytology (ASC-H and HSIL) were HPV16, HPV31, and HPV52. Within low-grade cytology (ASCUS and LSIL), HPV16, HPV66, and HPV56 were the most prevalent. Within glandular cytology (AGC-NOS and AGC-FN), the most prevalent genotypes were HPV18, HPV16 and HPV45.

The baseline prevalence of the different HPV genotypes by age group is presented in Table 1. A total of 732 (83.3%) women were positive for any HPV genotype, of which 644 (73.3%) were hrHPV-positive. HrHPV positivity declined with increasing age, from 79.5% among women under 30 years of age to 57.7% in women 45 years or older. The most prevalent HPV genotypes overall were HPV16 (29.1%, 256/879), HPV31 (9.2%, 81/879), and HPV52 (7.2%, 63/879), but in older women (≥45 years), HPV18 was the second-most common genotype. The overall prevalence of multiple HPV infections among HPV-positive women was 237/732 (32.4%), and it decreased significantly with age, being 93/209 (44.5%) in younger women (<30 years) and 39/143 (27.3%) in older women (≥45 years) (Table 1).

Approximately a quarter of the women under 45 years of age were current smokers (25.2% < 30 years, 26.4% 30–44 years), but only 18.4% smoked in the group of ≥45-year-old women (Table S1). Smokers tended to have more sex partners, and women with more partners tended to smoke. Former and current smokers among women under 45 years had an over twofold risk of being positive for HPV16 than nonsmokers. The corresponding ORs varied between 2.42 and 3.74 (95% CI range of 1.05–9.88) among women under 30 years old and between 2.51 and 3.29 (95% CI range of 1.35–6.40) among 30–44-year-old women. Smoking increased the risk of non-16-hrHPV among all women OR 1.76 (95% CI 1.14–2.72) but did not reach statistical significance when stratified according to age (Table 2). In women ≥45 years of age, 94.4% of smokers had smoked for 11 years or longer (Table S3). There were no statistically significant associations between smoking years and HPV16 prevalence in women ≥45 years compared to nonsmokers (Table S4). In women <30 years and 30–44 years, the same association between smoking years and increased prevalence of HPV16 was present and statistically significant.

The majority of the women in this study (62.2%) had experienced their sexual debuts at 16–19 years of age. The later in life the first intercourse was, the lower the prevalence of HPV16 infections was (Table 2). A later age for first intercourse was associated with lower HPV16 prevalence, with an OR of 0.56 (95% CI 0.37–0.85) for women with sexual debuts between 16–19 years compared with women with sexual debuts under the age of sixteen and an OR of 0.43 (95% CI 0.23–0.83) for women with sexual initiation at the age of 20 years or older. The prevalence of non-16-hrHPV or lrHPV, however, did not differ by age at first intercourse.

The lifetime number of partners was higher and the age at first intercourse was lower in younger women (<30 years) compared with older women (≥45 years). An increasing number of lifetime partners significantly increased the risk of overall hrHPV positivity (Table 2**)**. The increase in risk was highest for HPV16, with ORs ranging from 2.01 to 2.94 in the different age groups among women with 4–20 partners or more. Higher lrHPV prevalence was also significantly associated with multiple partners; however, this risk was not significantly increased statistically after 10 partners or more (Table 3). Younger women were more likely to practice oral sex than older women. However, the association between oral sex and HPV16 prevalence did not reach statistical significance when stratified according to age. The practice of anal sex was associated with increased prevalence of HPV16 infections in women under 30 years, with an OR of 3.56 (95%CI 1.36–9.35), but no significant association was observed with other hrHPV genotypes.

Almost half of the participants were nulliparous (45.5%), and only 13.8% had had three or more births. An increasing number of births was associated with lower prevalence of HPV16 compared with nulliparity overall (Table 2). Furthermore, decreased prevalence was also seen for non16-hrHPV among women with three or more births. The most popular contraceptive method was a hormonal intrauterine device (IUD) among older women and an estrogen and progestin combination of contraception among younger women.

Current smoking, together with the use of a hormonal IUD, increased the risk of non16-hrHPV among all women (*n* = 24), with an OR of 3.09 (95%CI 1.03–9.25) (data not shown). The use of vitamin D and atopia protected from HPV16 among women between ages 30–44, and drug abuse increased the risk of non16-hrHPV among all women. Termination of pregnancy, miscarriages, more than one partner within the last 12 months, and the use of alcohol were not associated with the prevalence of different HPV genotypes. Compared to ASCUS or being negative for intraepithelial lesion or malignancy (NILM), HSIL referral cytology among women under 45 years was strongly associated with the increased prevalence of HPV16 and non-16-hrHPV infections (Table S2). A similar but more modest association was seen with ASC-H cytology. For women 45 years or older, only HSIL cytology was associated with the prevalence of HPV16 infections. No other statistically significant associations were identified between the different referral cytologies and the non-16-hrHPV infections.

## 4. Discussion

In a well-screened Finnish colposcopy population, smoking among younger women associated more strongly with HPV16 than with other hrHPV genotypes. Older age at first intercourse seemed to protect more from HPV16 positivity than from other hrHPV genotypes. Interestingly, contraception had no significant association with the prevalence of HPV16.

While the association between lifestyle-related risk factors and HPV positivity have been well studied, the possible HPV genotype-specific differences in these risk factors have less frequently been explored. Organized screening has been in place since the 1960s in Finland, and thus, most women in our study population were well screened. We found HPV16 to be the most prevalent genotype (29.1%) in the whole study population and in all age groups, followed by HPV31 and HPV52. This genotype distribution is in line with previously published data from Finland showing a slightly different HPV genotype profile in the screening population compared with other countries in Europe [15], where HPV51 ranks third after HPV16 and HPV31 [2].

The rate of smokers in our study population (24.1%) was higher than in the general population (13%) in Finland [16]. Smoking at a younger age predicted HPV16 positivity rather than positivity for other hrHPV types. After stratification by the number of lifetime partners, the increased risk of HPV16 positivity due to current smoking was the highest in women with 1–3 lifetime partners. Among women with more than four lifetime sex partners, the association between smoking and HPV16 positivity was less pronounced, as the high number of lifetime sex partners alone explained the increased HPV16 positivity and, therefore, blurred the impact of smoking. Contraception or age at first intercourse did not affect the positive association of smoking with HPV16. Thus, smoking was an independent risk factor for HPV16 positivity. Smoking is a known independent risk factor for persistent HPV infection [17].

Surprisingly, among older women, no significant association between smoking and HPV16 positivity was found. This result was likely related to the fact that in the oldest age group, there were less smokers, and HPV16 was less prevalent. While the increasing number of smoking years significantly increased the risk of HPV16 in the two youngest age groups, the risk of HPV16 was not increased in the oldest age group with increased smoking years. Smoking affects the immune system and increases the risk of acquiring new HPV infections, as well as the risk of HPV persistence [18,19,20]. Of note, young smoking women have been found to not produce antibodies for HPV16 or maintain them [18]. Nevertheless, this influence of smoking on the early phase of HPV infections is not as obvious and not as well studied as the role of smoking in cervical carcinogenesis as a co-factor [20]. In our study, the rate of HPV16 positivity was increased among young former smokers as well. This could be explained by the long-term impact of smoking on the immune response mechanisms in cervical tissue. The risk of cervical cancer is known to be elevated among former smokers for up to 10 years after quitting smoking, but it decreases gradually thereafter [17].

An early age at first intercourse is considered a risk factor of cervical HSIL [8]. Interestingly, in our study, HPV16 was more prevalent among women with early sexual initiation than among women with a later sexual debut. Stratification according to the number of lifetime partners did not change the result. The risk of colposcopy may increase in women with HPV16 infection compared with other hrHPV genotypes because of the higher carcinogenic potential of HPV16. In a recent study in women vaccinated against HPV16 and 18, an increased age at first intercourse carried a higher risk for hrHPV other than HPV16 or 18 [21]. A previous study from Brazil also showed that an early sexual debut (<16 years) in women with abnormal cytology was significantly associated with HPV16 or 18 but not with other genotypes [22]. It has been proposed that the age of acquiring persistent HPV infection leading to cervical cancer is earlier for HPV16 than for other hrHPV genotypes [23]. Moreover, an early age at first intercourse has been linked to risk-taking behavior concerning HPV transmission (e.g., increased number of lifetime and recent sex partners, smoking, and binge drinking) [24]. Similarly, in the current study, an early sexual debut was associated with smoking, an increased number of partners, and binge drinking among all women (data not shown).

The relationship between the number of lifetime sex partners and HPV infections has been reported consistently in the literature [3,5,8], and with an increasing number of partners, the risk for hrHPV positivity is heightened [8]. Similarly, in a group of HPV16- and 18-vaccinated women, an increased number of partners enhanced the risk acquiring hrHPV genotypes other than HPV16 or 18. However, a high number of partners was not associated with persistence or progression to cervical intraepithelial neoplasia (CIN) 2 [21]. CIN2 and CIN3 are both high-grade precancerous lesions. Although their ability to progress if left untreated and regress spontaneously differ, both are considered precursors of cervical cancer. Our study showed a strong association between an increased number of sex partners and both HPV16 and non16-hrHPV, particularly among young women. This could be interpreted as a sexually active lifestyle and consequently frequent HPV transmissions reflecting the high incidence of HPV infections among young women in general [4], as well as HPV persistence, due to the setting here.

A clear difference was observed between the youngest and oldest age groups in the number of sex partners and in the age of sexual debut. Younger women had more partners and earlier initiation, which could refer to behavioral changes between generations. This result is consistent with data obtained in earlier population-based studies from Finland and Britain, where HPV16 seroprevalence was found to have increased, the number of sex partners was higher, and the recorded age at first intercourse was lower among younger women [25,26].

Previous meta-analyses have demonstrated that the use of an IUD was protective for cervical cancer compared with never using an IUD [27,28]. Furthermore, current IUD use has not been associated with the acquisition or persistence of an HPV infection [29]. However, a study from the US found significant association of CIN2+ with hormonal IUD use in the past 18 months, while the risk of CIN3+ was not increased [30]. In other words, the association was found only with CIN2, which has lower potential to progress and often regresses. In the present study, women using hormonal IUD had a higher prevalence of non16-hrHPV compared with women using condom contraception. When stratified according to the number of lifetime partners and smoking, non16-hrHPV was elevated only among those who had more than 11 lifetime partners or were current smokers, suggesting that there are other risk factors contributing to non16-hrHPV than the method of contraception per se. In many [9,31,32] but not all [7,33] studies, oral contraceptive use has been associated with HSIL and with HPV16 infection [34]. In our cohort, no significant association with HPV positivity was observed for estrogen and progestin contraceptive use. However, estrogen and progestin contraceptive use was significantly associated with HPV16 when compared with women with no contraception. One explanation could be that women using no contraception either live in a stable relationship, practice celibacy, or wish to get pregnant.

A high number of births (≥3) had a protective effect against all hrHPV genotypes (and an increasing number of births against HPV16). In contrast to our work, a previous study found an increased risk for HSIL in women with higher numbers of (full-term) pregnancies [31]. HrHPV prevalence altogether has been found to be higher in the Finnish screening population among nulliparous women (12%) compared with parous women (5–6%) [35]. The same phenomenon was reported from the UK among women with low-grade cytology; parous women had a reduced risk for hrHPV compared with nulliparous women [36]. The fact that none of the women with three or more children were younger than thirty in our study population points to the possibility that young women with a high prevalence of HPV infections may not have had the opportunity to give birth yet.

This study was carried out among the patient population of the colposcopy clinic in Helsinki, which is large, with over 4000 colposcopies performed per year. We were not able to take into account the incident character of HPV infections. However, we assume that the infections identified here represent persistent rather than transient infections, as our data consisted of women with a high frequency of high-grade cytology. Questionnaire-based data are susceptible to recall bias, and answers to some points in the questionnaire were missing. However, we conducted a substantial questionnaire for a well-defined group of women who eagerly and on a voluntary basis accepted to participate in this study to firmly expose the background information. Thus, possible differences in recall bias according to HPV genotype are unlikely.

## 5. Conclusions

The carcinogenic potentials of the different hrHPV genotypes, as well as their prevalence in different ages, vary. HPV infections are affected by various background factors, and these appear to differ between the different HPV genotypes. Younger smokers and women with sexual debuts at early ages were more likely to be positive for HPV16 than for other hrHPV genotypes. The methods of contraception, however, did not seem to have a clear effect on high-risk HPV-positivity regardless of the genotype. Further studies on the modifiable risk factors for the different HPV genotypes are still warranted for better targeting of possible interventions.

## Figures and Tables

**Table 1:** Baseline HPV genotype prevalence among 879 women referred to colposcopy in Helsinki, Finland.

	<30 y	30–44 y	≥45 y	All Women
	n (%)	n (%)	n (%)	n (%)
**High-Risk HPV Genotypes**	(*n* = 234)	(*n* = 444)	(*n* = 201)	(*n* = 879) *p* ^4^
16	90 (38.5)	136 (30.6)	30 (14.9)	256 (29.1) **0.000**
18	15 (6.4)	27 (6.1)	14 (7.0)	56 (6.4) 0.913
31	25 (10.7)	47 (10.6)	9 (4.5)	81 (9.2) **0.022**
33	14 (6.0)	16 (3.6)	7 (3.5)	37 (4.2) 0.302
35	5 (2.1)	8 (1.8)	4 (2.0)	17 (1.9) 0.949
39	11 (4.7)	14 (3.2)	8 (4.0)	33 (3.8) 0.571
45	7 (3.0)	23 (5.2)	4 (2.0)	34 (3.9) 0.133
51	24 (10.3)	22 (5.0)	13 (6.5)	59 (6.7) **0.032**
52	21 (9.0)	33 (7.4)	9 (4.5)	63 (7.2) 0.173
56	10 (4.3)	31 (7.0)	11 (5.5)	52 (5.9) 0.369
58	8 (3.4)	17 (3.8)	5 (2.5)	30 (3.4) 0.781
59	6 (2.6)	14 (3.2)	5 (2.5)	25 (2.8) 0.896
66	19 (8.1)	26 (5.9)	11 (5.5)	56 (6.4) 0.434
68	4 (1.7)	10 (2.3)	9 (4.5)	23 (2.6) 0.174
**Low-Risk HPV Genotypes**				
6	8 (3.4)	5 (1.1)	1 (0.5)	14 (1.6) **0.042**
11	0 (0.0)	2 (0.5)	1 (0.5)	3 (0.3) 0.611
30	0 (0.0)	1 (0.2)	0 (0.0)	1 (0.1) 1.000
40	0 (0.0)	0 (0.0)	0 (0.0)	0 (0.0) -
42	9 (3.8)	11 (2.5)	9 (4.5)	29 (3.3) 0.345
43	2 (0.9)	3 (0.7)	0 (0.0)	5 (0.6) 0.611
53	11 (4.7)	13 (2.9)	10 (5.0)	34 (3.9) 0.330
61	0 (0.0)	1 (0.2)	1 (0.5)	2 (0.2) 0.476
67	7 (3.0)	8 (1.8)	3 (1.5)	18 (2.0) 0.522
69	1 (0.4)	4 (0.9)	1 (0.5)	6 (0.7) 0.874
70	3 (1.3)	17 (3.8)	8 (4.0)	28 (3.2) 0.137
73	1 (0.4)	1 (0.2)	1 (0.5)	3 (0.3) 0.796
74	4 (1.7)	2 (0.5)	1 (0.5)	7 (0.8) 0.215
81	4 (1.7)	2 (0.5)	2 (1.0)	8 (0.9) 0.262
83	3 (1.3)	3 (0.7)	2 (1.0)	8 (0.9) 0.651
86	0 (0.0)	2 (0.5)	2 (1.0)	4 (0.5) 0.266
87	9 (3.8)	10 (2.3)	3 (1.5)	22 (2.5) 0.274
89	4 (1.7)	2 (0.5)	3 (1.5)	9 (1.0) 0.179
90	13 (5.6)	1 (0.2)	3 (1.5)	17 (1.9) **0.000**
91	5 (2.1)	8 (1.8)	2 (1.0)	15 (1.7) 0.699
**Different HPV Groups**				
Any HPV+	209 (89.3)	380 (85.6)	143 (71.1)	732 (83.3) **0.000**
HPV-negative	25 (10.7)	64 (14.4)	58 (28.9)	147 (16.7) **0.000**
High-risk HPV+ ^1^	186 (79.5)	342 (77.0)	116 (57.7)	644 (73.3) **0.000**
Low-risk HPV+ ^2^	66 (28.2)	82 (18.5)	48 (23.9)	196 (22.3) **0.013**
Single HPV	116 (55.5)	275 (72.4)	104 (72.7)	495 (67.6) **0.000**
Multiple HPV (≥2+) ^3^	93 (44.5)	105 (27.6)	39 (27.3)	237 (32.4) **0.000**

HPV genotypes are shown among all women and in age groups of <30, 30–44, and ≥45 years old. At the end of the table, multiple HPV findings are shown in the same age groups. Due to multiple infections, the total of low-risk and high-risk infections differs from the number of Any HPV+. ^1^ High-risk HPV types: 16, 18, 31, 33, 35, 39, 45, 51, 52, 56, 58, 59, 66, and 68. ^2^ Low-risk HPV types: 6, 11, 30, 40, 42, 43, 53, 61, 67, 69, 70, 73, 74, 81, 83, 86, 87, 89, 90, and 91. ^3^ Multiple infections defined as having two or more HPV genotypes detected simultaneously. ^4^ Chi-square and Fischer’s exact test. Statistically significant associations are shown in bold.

**Table 2:** Association between HPV positivity * and the different recorded risk factors among 879 women referred to colposcopy in Finland.

Recorded Risk Factors OR (95% CI)	HPV 16 vs. lr-HPV/HPV-Negative				nonHPV16-hrHPV vs. lrHPV/HPV-Negative			
	<30 y	30–44 y	≥45 y	All Women	<30 y	30–44 y	≥45 y	All Women
Contraception								
Condom	1.00	1.00	1.00	1.00	1.00	1.00	1.00	1.00
No	1.85 (0.59–5.75)	0.72 (0.35–1.48)	2.26 (0.67–7.60)	0.80 (0.50–1.30)	2.08 (0.65–6.70)	0.94 (0.49–1.79)	1.39 (0.63–3.08)	1.01 (0.65–1.56)
E+P ^1^	1.12 (0.47–2.69)	1.56 (0.69–3.56)	1.38 (0.12–15.72)	1.65 (0.97–2.83)	1.67 (0.69–4.05)	1.69 (0.78–3.66)	0.73 (0.12–4.53)	1.65 (0.99–2.74)
Progestin ^2^	1.83 (0.54–6.13)	0.94 (0.33–2.64)	3.67 (0.46–29.42)	1.60 (0.79–3.24)	2.17 (0.63–7.47)	0.93 (0.35–2.45)	0.98 (0.15–6.58)	1.34 (0.67–2.65)
Hormonal IUD	1.30 (0.21–8.03)	1.06 (0.49–2.28)	2.75 (0.61–12.48)	1.12 (0.62–2.04)	5.00 (0.96–25.94)	1.25 (0.62–2.54)	**3.81 (1.43**–**10.17)**	**1.98 (1.17**–**3.35)**
Cu IUD	0.65 (0.04–11.24)	1.00 (0.32–3.16)	NP^a^	0.99 (0.37–2.63)	1.67 (0.14–20.23)	0.53 (0.16–1.78)	0.73 (0.06–8.83)	0.74 (0.28–1.95)
Oral sex	1.40 (0.30–6.53)	0.87 (0.34–2.23)	2.32 (0.73–7.39)	**1.96 (1.10**–**3.52)**	3.13 (0.51–19.42)	0.82 (0.35–1.95)	1.11 (0.56–2.21)	1.37 (0.85–2.23)
Anal sex	**3.56 (1.36**–**9.35)**	0.78 (0.45–1.36)	0.85 (0.28–2.56)	1.28 (0.85–1.92)	2.24 (0.84–5.93)	0.74 (0.44–1.23)	1.51 (0.73–3.13)	1.12 (0.77–1.64)
Parity								
0	1.00	1.00	1.00	1.00	1.00	1.00	1.00	1.00
1–2	0.70 (0.30–1.62)	0.90 (0.51–1.59)	1.30 (0.37–4.55)	**0.61 (0.41**–**0.90)**	0.69 (0.30–1.59)	1.17 (0.70–1.96)	1.30 (0.55–3.07)	0.92 (0.64–1.31)
≥3	NP^b^	0.90 (0.43–1.88)	1.25 (0.33–4.70)	**0.48 (0.29**–**0.81)**	NP^b^	0.58 (0.28–1.20)	0.81 (0.31–2.07)	**0.48 (0.29**–**0.78)**
Alcohol use								
No	1.00	1.00	1.00	1.00	1.00	1.00	1.00	1.00
<6 doses/use	2.86 (0.42–19.65)	1.87 (0.63–5.55)	0.47 (0.12–1.89)	1.07 (0.52–2.22)	2.86 (0.42–19.65)	1.60 (0.63–4.09)	1.44 (0.44–4.77)	1.47 (0.78–2.79)
>6 doses/use	2.32 (0.78–6.86)	1.83 (0.73–4.57)	0.52 (0.15–1.82)	1.59 (0.87–2.91)	2.47 (0.83–7.30)	1.23 (0.57–2.67)	1.38 (0.45–4.26)	1.50 (0.87–2.57)
Smoking								
No	1.00	1.00	1.00	1.00	1.00	1.00	1.00	1.00
Yes	**3.74 (1.42**–**9.88)**	**3.29 (1.69**–**6.40)**	1.15 (0.35–3.81)	**2.94 (1.84**–**4.70)**	2.31 (0.88–6.07)	1.75 (0.94–3.26)	1.38 (0.61–3.15)	**1.76 (1.14**–**2.72)**
Ex-smoker	**2.42 (1.05**–**5.58)**	**2.51 (1.35**–**4.67)**	1.34 (0.53–3.34)	**2.02 (1.33**–**3.07)**	1.63 (0.72–3.71)	1.60 (0.91–2.81)	1.08 (0.55–2.11)	1.37 (0.94–2.00)
>1 partner in 12 months	0.88 (0.37–2.12)	0.63 (0.31–1.25)	1.71 (0.46–6.33)	0.96 (0.59–1.57)	1.20 (0.52–2.77)	0.93 (0.51–1.69)	1.14 (0.40–3.30)	1.16 (0.75–1.79)
Age at first intercourse								
≤15 y	1.00	1.00	1.00	1.00	1.00	1.00	1.00	1.00
16–19 y	**0.37 (0.17**–**0.81)**	1.17 (0.64–2.12)	**0.30 (0.11**–**0.86)**	**0.56 (0.37**–**0.85)**	0.69 (0.32–1.53)	1.24 (0.71–2.16)	0.63 (0.27–1.48)	0.84 (0.57–1.25)
≥20 y	0.59 (0.10–3.60)	0.62 (0.25–1.54)	0.56 (0.15–2.09)	**0.43 (0.23**–**0.83)**	0.77 (0.13–4.79)	0.81 (0.36–1.80)	0.74 (0.25–2.17)	0.69 (0.39–1.24)
Lifetime partners								
1–3	1.00	1.00	1.00	1.00	1.00	1.00	1.00	1.00
4–10	1.54 (0.51–4.66)	1.39 (0.62–3.14)	3.73 (0.96–14.51)	**2.01 (1.15**–**3.49)**	1.50 (0.50–4.54)	1.47 (0.70–3.08)	**2.40 (1.03**–**5.59)**	**1.83 (1.13**–**2.98)**
11–20	2.15 (0.66–7.01)	1.51 (0.64–3.55)	3.00 (0.65–13.76)	**2.36 (1.30**–**4.26)**	1.85 (0.56–6.07)	2.06 (0.95–4.46)	2.50 (0.96–6.50)	**2.42 (1.44**–**4.07)**
>20	**5.00 (1.03**–**24.28)**	2.20 (0.89–5.45)	2.86 (0.59–13.81)	**2.94 (1.55**–**5.58)**	**9.33 (2.00**–**43.63)**	1.55 (0.65–3.65)	2.57 (0.96–6.88)	**2.75 (1.54**–**4.88)**
TOP	1.40 (0.59–3.35)	0.95 (0.52–1.73)	0.65 (0.22–1.92)	1.00 (0.66–1.53)	1.07 (0.44–2.58)	0.94 (0.54–1.63)	0.74 (0.35–1.56)	0.91 (0.62–1.35)
Vitamin D usage	0.95 (0.47–1.93)	**0.49 (0.29**–**0.82)**	2.27 (0.91–5.68)	0.73 (0.51–1.05)	1.07 (0.53–2.15)	0.72 (0.44–1.18)	1.15 (0.63–2.10)	0.91 (0.66–1.27)
Atopia	0.83 (0.34–2.01)	**0.50 (0.26**–**0.97)**	1.99 (0.73–5.41)	0.79 (0.50–1.24)	0.98 (0.41–2.31)	0.76 (0.43–1.33)	1.17 (0.52–2.64)	0.93 (0.62–1.40)
Miscarriages	1.07 (0.26–4.49)	0.53 (0.28–1.02)	1.54 (0.56–4.29)	0.68 (0.42–1.12)	0.65 (0.14–3.04)	0.64 (0.36–1.13)	0.99 (0.44–2.22)	0.74 (0.48–1.14)
Drugs	1.36 (0.54–3.38)	1.25 (0.67–2.32)	NP^a^	1.47 (0.92–2.36)	1.67 (0.69–4.05)	1.12 (0.62–2.00)	2.14 (0.76–5.98)	**1.54 (1.00**–**2.37)**

Risk factor data were collected through a questionnaire. * HPV genotypes were grouped first into HPV16 vs. lrHPV/HPV-negatives and second group looking at non-16-hrHPV genotypes vs. lrHPV/HPV-negatives. HPV infection was recorded as a single infection or multiple infections. Risk factors were analyzed in all women and in age groups <30, 30–44, and ≥45 years old. ^1^ E+P includes estrogen and progestin pills and contraceptive patches and rings. ^2^ Progestin includes progestin pills and implants. Statistically significant associations are shown in bold. Cu-IUD = copper-releasing intrauterine device; HrHPV = high-risk human papillomavirus; Hormonal IUD = levonorgestrel-releasing IUD; LrHPV = low-risk HPV; NP^a^ = Non-pertinent due to perfect prediction; NP^b^ = Non-pertinent due to zero observation; and TOP = termination of pregnancy.

**Table 3:** Association between low-risk HPV infection and risk factors among women referred to colposcopy in Finland.

Recorded Risk Factors OR (95% CI)	lrHPV (Including Low-Risk Multiple Infections ^3^) vs. HPV-Negative			
	<30 y	30–44 y	≥45 y	All Women
Contraception				
Condom	1.00	1.00	1.00	1.00
No	0.44 (0.06–3.16)	0.55 (0.17–1.76)	1.33 (0.42–4.21)	0.73 (0.36–1.47)
E+P ^1^	1.09 (0.28–4.33)	1.75 (0.46–6.65)	NP^a^	1.50 (0.66–3.45)
Progestin ^2^	0.22 (0.02–2.45)	1.05 (0.21–5.19)	NP^a^	0.56 (0.16–1.93)
Hormonal IUD	0.88 (0.05–16.74)	1.56 (0.47–5.10)	1.78 (0.37–8.59)	1.38 (0.57–3.31)
Cu IUD	NP^a^	0.88 (0.14–5.51)	2.67 (0.14–49.76)	0.85 (0.19–3.67)
Oral sex	1.91 (0.16–22.63)	4.42 (0.52–37.44)	0.83 (0.30–2.28)	1.50 (0.68–3.31)
Anal sex	2.42 (0.40–14.69)	1.17 (0.50–2.71)	1.02 (0.31–3.30)	1.18 (0.64–2.18)
Parity				
0	1.00	1.00	1.00	1.00
1–2	1.12 (0.30–4.13)	1.03 (0.42–2.54)	1.08 (0.29–4.09)	0.85 (0.47–1.54)
≥3	NP^b^	1.93 (0.62–5.95)	1.39 (0.34–5.60)	1.13 (0.55–2.32)
Alcohol use				
No	1.00	1.00	1.00	1.00
<6 doses/use	1.00 (0.05–22.18)	0.70 (0.15–3.37)	0.62 (0.23–1.68)	0.62 (0.23–1.68)
>6 doses/use	0.90 (0.20–4.14)	0.83 (0.24–2.88)	0.81 (0.17–3.79)	0.82 (0.37–1.85)
Smoking				
No	1.00	1.00	1.00	1.00
Yes	1.43 (0.27–7.55)	1.25 (0.43–3.59)	1.67 (0.48–5.81)	1.36 (0.66–2.80)
Ex-smoker	0.77 (0.20–2.98)	0.91 (0.34–2.40)	0.85 (0.30–2.42)	0.81 (0.44–1.52)
>1 partner in 12 mo	1.85 (0.45–7.65)	0.89 (0.32–2.46)	3.19 (0.66–15.39)	1.52 (0.75–3.09)
Age at first intercourse				
≤15 y	1.00	1.00	1.00	1.00
16–19 y	0.89 (0.24–3.32)	0.88 (0.35–2.22)	1.21 (0.29–5.06)	0.89 (0.47–1.72)
≥20 y	1.00 (0.05–19.96)	0.77 (0.21–2.88)	1.67 (0.29–9.45)	0.90 (0.35–2.28)
Lifetime partners				
1–3	1.00	1.00	1.00	1.00
4–10	2.54 (0.42–15.21)	2.03 (0.56–7.40)	**7.00 (1.72–28.54)**	**3.50 (1.53–8.04)**
11–20	4.80 (0.68–33.80)	2.23 (0.57–8.69)	2.33 (0.45–12.23)	**3.10 (1.26–7.60)**
≥20	6.00 (0.34–107.42)	2.28 (0.52–9.99)	2.80 (0.52–14.96)	**2.75 (1.01–7.48)**
TOP	0.84 (0.20–3.62)	2.25 (0.90–5.64)	0.90 (0.30–2.67)	1.32 (0.71–2.46)
Vitamin D usage	0.64 (0.20–2.03)	1.22 (0.52–2.85)	0.84 (0.34–2.11)	0.90 (0.53–1.54)
Atopia	0.67 (0.16–2.75)	0.74 (0.28–1.92)	1.49 (0.44–5.08)	0.91 (0.47–1.76)
Miscarriages	2.29 (0.19–27.05)	1.33 (0.54–3.30)	0.31 (0.06–1.48)	0.87 (0.44–1.74)
Drugs	2.04 (0.43–9.70)	1.05 (0.39–2.81)	1.08 (0.19–6.29)	1.30 (0.63–2.70)

Risk factors were analyzed in all women and in age groups of <30, 30–44, and ≥45 years old. ^1^ E+P includes estrogen and progestin pills and contraceptive patches and rings. ^2^ Progestin includes progestin pills and implants. ^3^ Multiple infections defined as having two or more HPV genotypes recorded simultaneously. Statistically significant associations are shown in bold. Cu IUD = copper-releasing intrauterine device; HrHPV = high-risk human papillomavirus; Hormonal IUD = levonorgestrel-releasing IUD; LrHPV = low-risk human papillomavirus; NP^a^ = Non-pertinent due to perfect prediction; NP^b^ = Non-pertinent due to zero observation; and TOP = termination of pregnancy.

## Data Availability

The data presented in this study are available on request from the corresponding author. The data are not publicly available due to privacy reasons.

## Supplementary Materials

The following are available online at https://www.mdpi.com/article/10.3390/microorganisms9040750/s1. Figure S1: Data of different recorded risk factors of 879 women <30, 30–44, and ≥45 years referred to colposcopy. Table S2: Association between referral cytology and HPV infection among 879 women referred to colposcopy in Finland.
