# Supplementary material for: Distribution of HPV Genotypes Differs Depending on Behavioural Factors among Young Women
Source: Microorganisms. 2021 Apr 2;9(4):750. doi: 10.3390/microorganisms9040750 (PMC8066411; doi:10.3390/microorganisms9040750)
Supplement: Supplementary file 1 [file microorganisms-09-00750-s001.zip › Table S4 2021-3-23 Submission.pdf]

Table S4. Data of smoking years among currently smoking and former smoking women ≥45 years old referred to colposcopy in Finland.

**Smoking status**	**Smoker**	**Ex-smoker**
	**n(%)**	**n(%)**
Smoking years		
≤5	0(0.0)	18(27.3)
6-10	2(5.6)	19(28.8)
11-20	9(25.0)	18(27.3)
>20	25(69.4)	11(16.7)
	36(100.0)	66(100.0)
