# Supplementary material for: Distribution of HPV Genotypes Differs Depending on Behavioural Factors among Young Women
Source: Microorganisms. 2021 Apr 2;9(4):750. doi: 10.3390/microorganisms9040750 (PMC8066411; doi:10.3390/microorganisms9040750)
Supplement: Supplementary file 1 [file microorganisms-09-00750-s001.zip › Table S1 2021-04-02.pdf]

Table S1. Data of different recorded risk factors of 879 women <30, 30-44 and ≥45 years referred to colposcopy.

	<30y n(%)	30-44y n(%)	≥45y n(%)	All women n(%)
	234(26.6)	444(50.5)	201(22.9)	879(100.0)
Contraception				
No	38(16.3)	93(21.1)	93(48.2)	223(25.8)
Condom	56(24.0)	139(31.6)	41(21.5)	236(27.3)
E+P[1]	85(36.5)	75(17.1)	7(3.7)	167(19.3)
Progestin[2]	32(13.7)	32(7.3)	7(3.7)	71(8.2)
Hormonal-IUD	18(7.7)	81(18.4)	41(21.5)	140(16.2)
Cu-IUD	4(1.7)	20(4.6)	3(1.6)	27(3.1)
	233(100.0)	440(100.0)	191(100.0)	864(100.0)
Hormonal contraception				
No	98(42.1)	252(57.3)	136(71.2)	486(56.3)
Yes	135(57.9)	188(42.7)	55(28.8)	378(43.8)
	233(100.0)	440(100.0)	191(100.0)	864(100.0)
Oral sex				
Never	9(3.9)	39(8.9)	48(24.2)	96(11.1)
Ever/Regularly	223(96.1)	397(91.1)	150(75.8)	770(88.9)
	232(100.0)	436(100.0)	198(100.0)	866(100.0)
Anal sex				
Never	171(74.7)	306(70.0)	155(78.3)	632(73.1)
Ever/Regularly	58(25.3)	131(30.0)	43(21.7)	232(26.9)
	229(100.0)	437(100.0)	198(100.0)	864(100.0)
Parity				
0	187(79.9)	182(41.0)	31(15.4)	400(45.5)
1-2	47(20.1)	200(45.0)	111(55.2)	358(40.7)
≥3	0(0.0)	62(14.0)	59(29.4)	121(13.8)
	234(100.0)	444(100.0)	201(100.0)	879(100.0)
Use of alcohol				
no	22(9.5)	40(9.1)	19(9.6)	81(9.3)
<6 doses/use	12(5.2)	74(16.9)	57(28.6)	143(16.5)
>6doses/use	197(85.3)	324(74.0)	123(61.8)	644(74.2)
	231(100.0)	438(100.0)	199(100.0)	868(100.0)
Smoking				
No	103(44.0)	188(42.3)	94(46.8)	385(43.8)
Yes	59(25.2)	117(26.4)	37(18.4)	213(24.2)
Ex	72(30.8)	139(31.3)	70(34.8)	281(32.0)
	234(100.0)	444(100.0)	201(100.0)	879(100.0)
>1 partner in 12mo				
0-1	181(78.4)	360(82.6)	179(90.4)	720(83.2)
>1	50(21.6)	76(17.4)	19(9.6)	145(16.8)
	231(100.0)	436(100.0)	198(100.0)	865(100.0)

	<30y n(%)	30-44y n(%)	≥45y n(%)	All women n(%)
	234(26.6)	444(50.5)	201(22.9)	879(100.0)
Age at 1st intercourse				
≤15y	84(36.2)	115(26.1)	36(17.9)	235(26.9)
16-19y	138(59.5)	276(62.7)	132(65.7)	546(62.5)
≥20y	10(4.3)	49(11.1)	33(16.4)	92(10.5)
	232(100.0)	440(100.0)	201(100.0)	873(100.0)
Lifetime partners #				
1-3	24(10.3)	54(12.4)	39(19.7)	117(13.4)
4-10	97(41.8)	161(37.1)	80(40.4)	338(38.7)
11-20	65(28.0)	135(31.1)	42(21.2)	242(27.7)
>20	46(19.8)	84(19.4)	37(18.7)	167(19.1)
	232(100.0)	434(100.0)	198(100.0)	873(100.0)
TOP				
No	184(78.6)	339(76.4)	160(79.6)	683(77.7)
Yes	50(21.4)	105(23.6)	41(20.4)	196(22.3)
	234(100.0)	444(100.0)	201(100.0)	879(100.0)
Vitamine-D usage				
No	123(53.0)	191(43.2)	82(41.0)	396(45.3)
Yes	109(47.0)	251(56.8)	118(59.0)	478(54.7)
	232(100.0)	442(100.0)	200(100.0)	874(100.0)
Atopia				
No	185(80.4)	357(81.0)	163(81.9)	705(81.0)
Yes	45(19.6)	84(19.0)	36(18.1)	165(19.0)
	230(100.0)	441(100.0)	199(100.0)	870(100.0)
Miscarriages				
No	221(94.4)	360(81.1)	166(82.6)	747(85.0)
Yes	13(5.6)	84(18.9)	35(17.4)	132(15.0)
	234(100.0)	444(100.0)	201(100.0)	879(100.0)
Drugs				
Never	182(78.1)	342(77.4)	183(91.0)	707(80.7)
Yes/tried	51(21.9)	100(22.6)	18(9.0)	169(19.3)
	233(100.0)	442(100.0)	201(100.0)	876(100.0)
Referral cytology				
ASCUS	28(12.0)	28(6.3)	23(11.4)	79(9.0)
LSIL	55(23.5)	182(41.0)	88(43.8)	325(37.0)
ASC-H	67(28.6)	99(22.3)	40(19.9)	206(23.4)
HSIL	59(25.2)	90(20.3)	26(12.9)	175(19.9)
AGC-NOS	4(1.7)	9(2.0)	10(5.0)	23(2.6)
AGC-FN	3(1.3)	8(1.8)	7(3.5)	18(2.0)
NILM	0(0.0)	1(0.2)	3(1.5)	4(0.5)
Not taken	18(7.7)	27(6.1)	4(2.0)	49(5.6)

	234(100.0)	444(100.0)	201(100.0)	879(100.0)

[1]E+P includes estrogen and progestin pills, contraceptive patches and rings

[2]Progestin includes progestin pills and implants

Cu-IUD = copper-releasing intrauterine device, Hormonal-IUD = Levonorgestrel-releasing IUD, HrHPV = high-risk human papillomavirus, LrHPV = low-risk human papillomavirus, NP[a] = Non pertinent due to perfect prediction, NP[b] = Non pertinent due to zero observation, TOP = termination of pregnancy, NILM = negative for intraepithelial lesion or malignancy, AS-CUS = atypical squamous cells of undetermined significance, LSIL = low-grade squamous intraepithelial lesion, HSIL = high-grade squamous intraepithelial lesion, ASC-H = atypical squamous cells cannot exclude HSIL, AGC-NOS = atypical glandular cells not otherwise specified, AG-FN = atypical glandular cells that favor neoplasia.
