# Supplementary material for: Distribution of HPV Genotypes Differs Depending on Behavioural Factors among Young Women
Source: Microorganisms. 2021 Apr 2;9(4):750. doi: 10.3390/microorganisms9040750 (PMC8066411; doi:10.3390/microorganisms9040750)
Supplement: Supplementary file 1 [file microorganisms-09-00750-s001.zip › Table S2 2021-04-02.pdf]

Table S2. Association between referral cytology and HPV infection among 879 women referred to colposcopy in Finland.

**Referral cytology OR (95% CI)**	**HPV 16 vs. lrHPV/HPV-negative**			
	**<30 y**	**30-44y**	**≥45y**	**All women**
ASCUS+NILM	1.00	1.00	1.00	1.00
LSIL	1.39(0.43-4.49)	1.68(0.54-5.20)	1.39(0.33-5.77)	1.41(0.73-2.75)
ASC-H	**3.50(1.14-10.74)**	**7.03(2.10-23.50)**	1.76(0.37-8.32)	**4.25(2.13-8.49)**
HSIL	**12.75(3.24-50.12)**	**23.04(5.73-92.66)**	**7.50(1.49-37.66)**	**14.79(6.66-32.85)**
AGC-NOS	NP[a]	0.40(0.04-4.24)	0.71(0.06-8.15)	0.33(0.07-1.59)
AGC-FN	NP[a]	0.80(0.07-9.67)	NP[a]	0.70(0.13-3.72)
AGC-NOS/FN	0.75(0.06-9.72)	0.53(0.08-3.40)	0.45(0.04-4.98)	0.44(0.13-1.49)
Not taken	2.25(0.48-10.60)	1.00(0.23-4.37)	NP[a]	1.83(0.71-4.72)
	nonHPV16- hrHPVvs. lrHPV/HPV-negative			
	<30y	30-44y	≥45y	All women
ASCUS+NILM	1.00	1.00	1.00	1.00
LSIL	3.00(1.00-9.02)	1.94(0.81-4.63)	2.19(0.83-5.75)	**2.33(1.35-4.01)**
ASC-H	**3.38(1.10-10.38)**	**3.14(1.16-8.55)**	1.88(0.63-5.58)	**2.85(1.55-5.23)**
HSIL	**7.88(1.95-31.75)**	**7.40(2.16-25.31)**	3.44(0.92-12.79)	**6.41(3.06-13.43**)
AGC-NOS	1.50(0.17-12.94)	0.33(0.06-1.99)	0.54(0.09-3.21)	0.56(0.19-1.61)
AGC-FN	NP[a]	1.33(0.24-7.28)	1.41(0.25-7.90)	1.79(0.60-5.38)
AGC-NOS/FN	3.00(0.44-20.44)	0.67(0.18-2.46)	0.85(0.22-3.33)	0.95(0.42-2.15)
Not taken	3.00(0.67-13.40)	0.83(0.26-2.66)	NP[a]	1.83(0.81-4.12)
	lrHPV vs. HPV-negative			
	<30y	30-44y	≥45y	All women
ASCUS+NILM	1.00	1.00	1.00	1.00
LSIL	2.50(0.49-12.64)	3.00(0.73-12.40)	1.50(0.44-5.09)	1.80(0.85-3.84)
ASC-H	0.50(0.10-2.60)	0.82(0.13-5.08)	0.20(0.03-1.21)	0.42(0.16-1.13)
HSIL	1.00(0.10-9.61)	4.50(0.49-41.25)	NP[a]	0.80(0.23-2.80)
AGC-NOS	1.00(0.05-19.96)	NP[a]	0.25(0.02-2.64)	0.25(0.05-1.25)
AGC-FN	NP[b]	1.50(0.10-23.07)	NP[a]	0.27(0.03-2.44)
AGC-NOS/FN	1.00(0.05-19.96)	0.38(0.03-4.37)	0.15(0.02-1.50)	0.25(0.06-1.00)
Not taken	NP[a]	1.00(0.16-6.35)	NP[b]	0.37(0.09-1.51)

HPV genotypes were grouped into the following categories: 1) HPV 16 vs. lrHPV/HPV-negatives; 2) other hrHPV genotypes (excluding HPV 16) vs. lrHPV/HPV-negative group; and 3) lrHPV (including low-risk multiple HPV infections) vs. HPV-negative. Referral cytology was evaluated in all women and in different age group categories of: <30, 30-44 and ≥45 years old. Statistically significant associations are shown in bold.

HrHPV = high-risk human papillomavirus, LrHPV = low-risk human papillomavirus, NP[a] = Non pertinent due to perfect prediction, NP[b] = Non pertinent due to zero observation, NILM = negative for intraepithelial lesion or malignancy, AS-CUS = atypical squamous cells of undetermined significance, LSIL = low-grade squamous intraepithelial lesion, HSIL = high-grade squamous intraepithelial lesion, ASC-H = atypical squamous cells cannot exclude HSIL, AGC-NOS = atypical glandular cells not otherwise specified, AG-FN = atypical glandular cells that favor neoplasia.
