# Supplementary material for: Distribution of HPV Genotypes Differs Depending on Behavioural Factors among Young Women
Source: Microorganisms. 2021 Apr 2;9(4):750. doi: 10.3390/microorganisms9040750 (PMC8066411; doi:10.3390/microorganisms9040750)
Supplement: Supplementary file 1 [file microorganisms-09-00750-s001.zip › Table S3 2021_3_23 Submission.pdf]

Table S3. Association between HPV16 infection and smoking years among women referred to colposcopy in Finland.

Recorded risk factors	HPV16 vs. low-risk HPV/HPV-negative			
**OR (95% CI)**	**<30y**	**30-44y**	**≥45y**	**All women**
Smoking years				
No	1.00	1.00	1.00	1.00
<11y	**2.60(1.24-5.44)**	**2.91(1.51-5.59)**	2.08(0.73-5.89)	**2.92(1.90-4.49)**
≥11y	**8.42(1.00-70.64)**	**2.77(1.46-5.25)**	0.84(0.30-2.37)	**1.84(1.16-2.91)**

Smoking years where analysed in all women and in age groups of: <30, 30-44 and ≥45 years old.
